# Functional Recovery and Serum Angiogenin Changes According to Intensity of Rehabilitation Therapy After Stroke

**DOI:** 10.3389/fneur.2021.767484

**Published:** 2021-11-25

**Authors:** Nicolás Garcia-Rodriguez, Susana Rodriguez, Pedro Ignacio Tejada, Zuberoa Maite Miranda-Artieda, Natalia Ridao, Xavi Buxó, María Engracia Pérez-Mesquida, Maria Rosario Beseler, Juan B. Salom, Laura M. Pérez, Marco Inzitari, Sergio Otero-Villaverde, Rosa Martin-Mourelle, Mercedes Molleda, Manuel Quintana, Marta Olivé-Gadea, Anna Penalba, Anna Rosell

**Affiliations:** ^1^Neurovascular Research Laboratory, Vall d'Hebron Research Institute, Universitat Autònoma de Barcelona, Barcelona, Spain; ^2^Unidad de Rehabilitación Neurológica y Daño Cerebral, Hospital Vall d'Hebron, Barcelona, Spain; ^3^Unidad de Daño Cerebral del Hospital de Górliz, Osakidetza, Górliz, Spain; ^4^Servei de Medicina Física i Rehabilitació, Parc Taulí Hospital Universitari, Institut d'Investigació i Innovació Parc Taulí I3PT, Sabadell, Spain; ^5^Servicio de Medicina Física y Rehabilitación, Hospital Universitario y Politécnico La Fe, Valencia, Spain; ^6^Unidad Mixta de Investigación Cerebrovascular, Instituto de Investigación Sanitaria La Fe–Universitat de Valencia, Valencia, Spain; ^7^Departamento de Fisiología, Universidad de Valencia, Valencia, Spain; ^8^RE-FiT Barcelona Research Group, Vall d'Hebron Institute of Research, Parc Sanitari Pere Virgili, Barcelona, Spain; ^9^Parc Sanitari Pere Virgili, Area of Intermediate Care, Barcelona, Spain; ^10^Universitat Oberta de Catalunya, Barcelona, Spain; ^11^Hospital Marítimo de Oza, A Coruña, Spain; ^12^Hospital Universitari Germans Tries i Pujol, Badalona, Spain; ^13^Epilepsy Research Group and Epilepsy Unit, Vall d'Hebron Research Institute and Vall d'Hebron Hospital, Barcelona, Spain; ^14^Stroke Group, Vall d'Hebron Research Institute, Barcelona, Spain

**Keywords:** angiogenin, intensive therapy, rehabilitation, biomarker, recovery

## Abstract

**Background:** Rehabilitation is still the only treatment available to improve functional status after the acute phase of stroke. Most clinical guidelines highlight the need to design rehabilitation treatments considering starting time, intensity, and frequency, according to the tolerance of the patient. However, there are no homogeneous protocols and the biological effects are under investigation.

**Objective:** To investigate the impact of rehabilitation intensity (hours) after stroke on functional improvement and serum angiogenin (ANG) in a 6-month follow-up study.

**Methods:** A prospective, observational, longitudinal, and multicenter study with three cohorts: strokes in intensive rehabilitation therapy (IRT, minimum 15 h/week) vs. conventional therapy (NO-IRT, <15 h/week), and controls subjects (without known neurological, malignant, or inflammatory diseases). A total of seven centers participated, with functional evaluations and blood sampling during follow-up. The final cohort includes 62 strokes and 43 controls with demographic, clinical, blood samples, and exhaustive functional monitoring.

**Results:** The median (IQR) number of weekly hours of therapy was different: IRT 15 (15–16) vs. NO-IRT 7.5 (5–9), *p* < 0.01, with progressive and significant improvements in both groups. However, IRT patients showed earlier improvements (within 1 month) on several scales (CAHAI, FMA, and FAC; *p* < 0.001) and the earliest community ambulation achievements (0.89 m/s at 3 months). There was a significant difference in ANG temporal profile between the IRT and NO-IRT groups (*p* < 0.01). Additionally, ANG was elevated at 1 month only in the IRT group (*p* < 0.05) whereas it decreased in the NO-IRT group (*p* < 0.05).

**Conclusions:** Our results suggest an association of rehabilitation intensity with early functional improvements, and connect the rehabilitation process with blood biomarkers.

## Introduction

Stroke is a leading cause of disability, with more than 16.9 million people having a first stroke every year, 5.9 million stroke-related deaths, and a calculated loss of 102 million Disability-Adjusted Life-Years (1, 2). In the last decades, there has been a marked decrease in stroke mortality as a result of improved primary care interventions, better neuroimaging diagnosis, and improved stroke management (with specialized stroke units, thrombolytic, and endovascular treatments) (3). Beyond this, the evidence-based approach to achieve functional improvement in daily-life activities and reduce disabilities in stroke survivors has been implementing personalized rehabilitation programs with multidisciplinary teams working under the physiatrist's supervision (4). Recently, an interesting debate has taken place on the need to implement early rehabilitation interventions (first 24 h) which might worsen the outcome at high doses but might be beneficial with high frequency (5, 6), highlighting the need for a fine-tuning of the interventions. Additionally, other studies have demonstrated that high-intensity therapies with a larger amount of hours are determinant for a good prognosis (7, 8), although the standard time and dose to achieve improvements is planned individually (9, 10). This is the case for intensive rehabilitation therapy (IRT), defined as rehabilitation therapy of more than 15 h per week by a physical therapist, an occupational therapist, and/or a speech therapist, with close monitoring of patient progress to adjust the program (11). Other clinical evidence has shown that with high-quality, high-dose, high-intensity upper limb neurorehabilitation during a 3-week (90 h) program clinical improvements in upper limb deficits and activity can be achieved in chronic stroke patients starting more than 6 months after the event (12). And in a retrospective analysis comparing this cohort with a conventional low-intensity treatment cohort it is described that, despite responsiveness of both treatments, the high-intensity approach showed a consistent higher impact at all stages post-stroke (13).

Neural plasticity and vascular remodeling are assumed as the basis of post-stroke recovery as reported in preclinical models (14, 15). In this context, understanding the role of specific biomarkers in pathophysiological brain changes might serve as a bridge between the fundamental science and patients' clinical management, including monitoring rehabilitation goals and the duration of the intervention (16, 17). In this regard, we focused on angiogenin (ANG), a member of the ribonuclease superfamily that acts as a potent angiogenic protein triggering cell proliferation, migration, or survival (18, 19), which has been recently identified as a repair-associated factor in post-stroke rehabilitation by our group (15).

In this multicenter study we aimed at studying the influence of the rehabilitation therapy intensity received after stroke on the functional improvements, and for the first time studying the response of a blood biomarker in response to the dose of therapy received during post-stroke recovery.

## Materials and Methods

### Study Cohorts

This study comprises a cohort of 62 post-stroke patients recruited in two periods within the prospective, observational, longitudinal, and multicenter SMARRTS study (Studying Markers of Angiogenesis and Repair during Rehabilitation Therapy after Stroke): ischemic stroke patients under IRT (between February 2014 and May 2015) (15) and both ischemic and hemorrhagic stroke patients under IRT or conventional rehabilitation therapy (between February 2017 and June 2018) from seven Spanish hospitals. Inclusion criteria were: first-ever ischemic or hemorrhagic stroke, age ≤ 75 years, modified Rankin scale (mRS) ≤ 2 before stroke and mRS post-stroke from three to five, stable medical condition, and the signature of informed consent. The exclusion criteria were: previous stroke or transient ischemic attacks, malignant infarct, global aphasia, hemorrhage from arteriovenous malformations or cerebral aneurysms, previous cognitive decline, or recent infectious, inflammatory or malignant disease.

The study was approved by all the clinical research ethics committee sites [HUVH PR(IR)317/2013-PR(IR)346/2016, PI16/00981/CEI:PI-17-056, Comité de Ética de Investigación de A Coruña-Ferrol 2017-125, Corporació Sanitària Parc Taulí de Sabadell 2017521, Hospital Universitario Politécnico La Fe 2016/0727, Euskadi PI2016168]. Healthy subjects (43) without known neurological, malignant, infectious, or inflammatory diseases were enrolled as the control cohort. All subjects signed informed consent following the Declaration of Helsinki.

STROBE guidelines for reporting observational studies were followed in this study (20).

### Rehabilitation Interventions

All included patients followed a comprehensive rehabilitation program, including physiotherapy, occupational therapy, speech therapy, and/or neuropsychology, however, some centers offered intensive rehabilitation therapies whereas others followed more conventional programs. For the IRT program only subacute post-stroke patients with moderate/severe disabilities in two or more areas (gait, transfers, activities of daily living, swallowing and/or communication), with mRS previous to stroke ≤ 2 who can participate in a minimum of 3 h of comprehensive therapy per day (5 days/week) were included, according to institutional guidelines (21). When the clinical stroke condition was stable patients started mobilizations followed by a comprehensive rehabilitation program defined as IRT (≥15 h per week) or NO-IRT (conventional therapy with <15 h per week). For all centers, a physiatrist designed an initial rehabilitation program according to patients' impairments, and all patients were treated in inpatient rehabilitation units or day-hospital facilities. Rehabilitation continued until completion of a minimum of the proposed objectives or when functional stability was achieved. For further objectives, patients continued an outpatient rehabilitation program.

### Study Protocol and Functional Follow-Up

**Figure 1:**
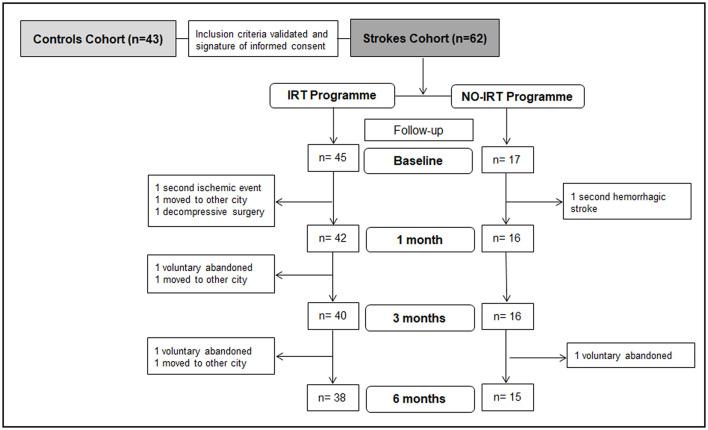
Study design. Scheme showing the studied cohorts and follow-up visits when a battery of tests to assess motor and functional status was conducted together with blood samples extraction. Controls were recruited in a unique inclusion visit when blood samples were obtained.

A total of 62 patients were initially included in the study. However, 3 of them voluntarily abandoned the study, and 6 were withdrawn from the study due to the following: decompressive cranial surgery (1), secondary aneurismal hemorrhagic stroke (1), a second ischemic event (1), or moving to other cities (3). This reduced the number of subjects in the follow-up analysis, as represented in the flow diagram in Figure 1. A baseline inclusion visit was conducted before the rehabilitation program started by an experienced physiatrist who collected demographic, clinical, and stroke-related data together with a battery of tests to assess motor and functional status (details in the Supplementary Methods). During the baseline interview, previous physical activity was considered as any cardiovascular exercise routine such as running, swimming, cycling, and obesity was calculated BMI > 29.

Follow-up visits were conducted by the same physiatrist at 1, 3, and 6 months after the start of rehabilitation including a battery of tests to assess neuro-functional status: the mRS (scores 0–6), the Granger modified Barthel Index (BI, scores 0–100) (22), the Fugl-Meyer Assessment score for the upper extremity (FMA, scores 0–66) (23), the Functional Ambulation Categories (FAC, scores 0–5), the Chedoke Arm and Hand Activity Inventory (CAHAI, scores 13–91) (24), the 10-m walk test (velocity is registered), the Medical Research Council scale (MRC, scores 0–5) of the upper and lower extremities at the proximal/distal level, and the Modified Asworth Scale (MAS, scores 0–4, including 1+) (25).

We also analyzed changes in the scores during follow-up visits vs. baseline scores to assess improvements in the neurological function: Rankin improvement was defined as a decrease of ≥1 point. For the FMA, the improvement was defined as an increase of ≥10 points, described previously as the minimal clinical important difference (26). For the Chedoke Arm and Hand Activity Inventory, an improvement was defined as an increase of ≥7 points (27). For the 10-m walk test, the walking velocity was calculated, and improvement was considered if walking velocity increased by >0.3 m/s. The FAC was categorized into three categories: cannot walk (score 0), dependent walk (scores 1–3), and independent walk (scores 4 and 5), and improvement was defined as a shift to a higher category (28). For the MRC scale, our analysis differentiated classification between normal (score 5) or impaired (scores 0–4) muscle strength.

At all visits blood sampling in serum-separating tubes was obtained, centrifuged at 1,500 rpm for 15 min, and serum stored at −80°C until use.

### Angiogenin Measurement

Serum levels of ANG were measured by ELISA (#DAN00; R&D Systems, USA) and analyzed together with previous-obtained results from cohort one using the same test (15). Briefly, 200 μl of diluted serum samples (1:200) were loaded per duplicate, and only values with a coefficient of variation < 20% were accepted for the statistical analysis. To verify low inter-plate variability (7 ELISA plates were analyzed in total) we included a commercial internal control from Sigma-Aldrich (Human serum type AB, male, from clotted, cat#H6914), and a coefficient of variation < 20% was accepted.

### Statistical Analysis

The SPSS 20.0 package was used for statistical analyses. The database entries were reviewed by an independent researcher. Descriptive statistics: categorical variables were reported as frequencies (percentages) and continuous variables as mean ± standard deviation (SD) or median [interquartile range (IQR)], as appropriate. Missing data were considered at random and no imputations were used. The normality assumption of quantitative variables was checked with the use of quantile-quantile (Q-Q) plots. Statistical significance was assessed by Pearson's chi-square or Fisher's exact test for categorical variables, Student's *t*-test for continuous variables, and the Mann-Whitney U test for functional scores and numerical variables without normal distribution. Temporal profile changes in normally distributed variables were analyzed with repeated measures ANOVA (Bonferroni *post-hoc* test) or the Friedman followed by Wilcoxon tests for non-normal distributions. Box plots were used to represent the temporal profile of non-normal distributed variables (functional scores) and bar-graphs showing mean with 95% confidence interval to represent normally distributed variables (temporal profile of ANG). Pearson (normal distribution) or Spearman (non-normal distribution) tests were used. Repeated measures in general linear models were conducted to assess changes in the ANG temporal profile between the two rehabilitation groups, controlling for other baseline characteristics (CAHAI). The temporal profile changes in the functional scales were analyzed with Mixed Models Analysis generated by the SAS System (Version W32_7 PRO) adjusting for the baseline scores. A *p*-value < 0.05 was considered statistically significant.

## Results

### Characteristics of the Study Cohorts at Baseline

The baseline characteristics of the stroke cohort and controls are described in Table 1. Of note, our stroke cohort presented more men (79.7 vs. 20.3%, *p* < 0.01), more tobacco users (37.1 vs. 16.7%, *p* = 0.02), and less previous exercise (39 vs. 76.7%, *p* < 0.001) than the control. Importantly, our two rehabilitation groups presented similar baseline characteristics except for the previous statins medication which was more frequent in the IRT group (46.7 vs. 17.6%, *p* = 0.03) as shown in Table 2.

**Table 1:** Baseline characteristics of the control and stroke cohorts.

	**Stroke cohort**	**Control cohort**	***p*-value**
	***n* = 62**	***n* = 43**	
Age (years)	57.59 ± 9.7	60.8 ± 10.8	0.11
Sex, males	79.7 (51)	20.3 (13)	**<0.01**
**Risk factors and comorbidities**			
Alcohol	27.4 (17)	23.8 (10)	0.65
Tobacco	37.1 (23)	16.7 (7)	**0.02**
Atrial fibrillation	9.7 (6)	0 (0)	0.07
Hypertension	61.3 (38)	46.5 (20)	0.16
Dyslipidemia	40.3 (25)	46.5 (20)	0.52
Diabetes mellitus	19.7 (12)	18.6 (8)	0.89
Obesity	29.5 (18)	34.9 (15)	0.56
Previous exercise	39.0 (23)	76.7 (33)	**<0.001**
Cardiopathy	6.5 (4)	0 (0.0)	0.14
Osteoarticular	12.9 (8)	32 (14)	**0.01**
Psychiatric	16.1 (10)	9.3 (4)	0.31
**Previous medication**			
Anti-platelets	21 (13)	14.3 (6)	0.38
Anti-coagulants	4.8 (3)	0 (0.0)	0.27
Statins	38.7 (24)	28.6 (12)	0.28
Anti-hypertensives	53.2 (33)	40.5 (17)	0.2
Anti-diabetic	17.7 (11)	14.3 (6)	0.64
**Angiogenin**			
Baseline levels (ng/mL)	520.8 ± 139.2	432.8 ± 155.7	**<0.01**

*Variables are expressed as a percentage (number of cases, n) or mean ± SD. Differences were assessed with a t-test, chi-square test, or Fisher exact test*.

*Statistically significant differences are highlighted in bold*.

**Table 2:** Baseline characteristics of the IRT vs. NO-IRT cohorts.

	**IRT**	**NO-IRT**	***p*-value**
	***n* = 45**	***n* = 17**	
Age	56.4 ± 9.1	60.6 ± 10.8	0.13
Sex, males	82.2 (37)	82.4 (14)	0.72
**Risk factors and comorbidities**			
Alcohol	28.9 (13)	23.5 (4)	0.72
Tobacco	40.0 (18)	29.5 (5)	0.53
Atrial fibrilation	8.9 (4)	11.8 (2)	0.64
Hypertension	66.7(30)	47.1 (8)	0.23
Dyslipidemia	46.7 (21)	23.5 (4)	0.09
Diabetes mellitus	25.0 (11)	5.9 (1)	0.15
Obesity	29.5 (13)	29.4 (5)	1
Previous exercise	38.1 (16)	41.2 (7)	0.8
Cardiopathy	11.8 (2)	4.4 (2)	0.3
Osteoarticular	8.9 (4)	23.5 (4)	0.18
Psychiatric	20 (9)	5.9 (1)	0.26
**Previous medication**			
Anti-platelets	22.2 (10)	17.6 (3)	1
Anticoagulants	4.4 (2)	5.9 (1)	1
Statins	46.7 (21)	17.6 (3)	**0.03**
Anti- hypertensives	55.6 (25)	47.1 (8)	0.58
Anti-diabetic	22.2(10)	5.9 (1)	0.26

*Variables are expressed as a percentage (number of cases) or mean ± SD. Differences were assessed with a t-test, chi-square test, or Fisher exact test*.

*Statistically significant differences are highlighted in bold*.

### Clinical Characteristics and Functional Outcome

The two rehabilitation groups presented similar clinical characteristics at emergency admission: stroke characteristics, acute treatment, hospitalization regimen, or baseline functional scores (see Tables 3, 4). Significant differences were only found in the number of rehabilitation hours per week: IRT 15 (15–16) vs. NO-IRT 7.5 (5–9), *p* < 0.01, and in the baseline CAHAI score which was lower in the IRT group: 13 (13–16) vs. 18.5 (13–82), *p* = 0.02.

**Table 3:** Clinical characteristics of IRT and NO IRT group on admission.

	**IRT**	**NO-IRT**	***p*-value**
	***n* = 45**	***n* = 17**	
Rankin	4.5 (4–5)	5 (4–5)	0.46
NIHSS	12 (7.5–17)	11 (6–14)	0.32
NIHSS (motor)	7 (4–9)	6.5 (5–11)	0.65
Stroke laterality, left	48.9 (22)	41.2 (7)	0.58
**Stroke type, ischemic**	73.3 (33)	70.6 (11)	0.82
**Vascular territory**			0.7
Carotid	78.8 (25)	83.3 (10)	
Vertebrobasilar	24.2 (8)	16.7 (2)	
**Ischemic etiology**			0.45
Cardioembolic	27.3 (9)	8.3 (1)	
Atherothrombotic	21.2 (7)	25 (3)	
Lacunar	18.2 (6)	41.7 (5)	
Others	12.1 (4)	8.3 (1)	
Undetermined	21.2(7)	16.7 (2)	
**OCSP classification**			0.35
TACI	46.9 (15)	27.3 (3)	
PACI	15.6 (5)	9.1 (1)	
LACI	25 (8)	54.5 (6)	
POCI	12.5 (4)	9.1 (1)	
**Acute treatment**			
Thrombolytic therapy	15.4 (4)	11.8 (2)	0.48
Endovascular treatment	6.7 (3)	17.6 (3)	0.33
Hemorrhagic transformation	4 (1)	0 (0)	1
**Stroke type, hemorrhagic**	26.7 (12)	29.4 (5)	0.53
**Location**			0.51
Deep	91.7 (11)	80 (4)	
Lobar	8.3 (1)	20 (1)	
**Hemorrhagic etiology**			0.33
Hypertensive	83.3 (10)	100 (5)	
Undetermined	16.7(2)	0 (0)	

*Variables are expressed as a percentage (number of cases) or median (IQR). OCSP, Oxfordshire Community Stroke Project; TACI, total anterior cerebral infarct; LACI, lacunar cerebral infarct; PACI, partial anterior cerebral infarct; POCI, posterior cerebral infarct. Differences were assessed with a t-test, Mann-Whitney-U, chi-square test, or Fisher exact test*.

**Table 4:** Post-stroke rehabilitation characteristics at baseline visit.

	**IRT**	**NO-IRT**	***p*-value**
	***n* = 45**	***n* = 17**	
**Hospitalization regimen**			
Inpatient rehabilitation	95.6 (43)	100 (17)	1
Day-hospital rehabilitation	4.4 (2)	0 (0)	1
Time stroke- RHB program in days	14 (9–19)	11 (8–14)	0.09
Time stroke-sample in days	14 (8.25–19)	10 (6–13.5)	0.08
RHB hour/week at baseline	15 (15–16)	7.5 (5–9)	**<0.01**
**Functional scores**			
Rankin	4 (3–5)	5 (3.2–5)	0.2
Barthel	35 (20–68)	23 (20–36.5)	0.12
NIHSS	9 (5–14)	9 (4–11)	0.38
CAHAI	13 (13–16)	18.5 (13–82)	**0.02**
FMA	8.5 (4–40.7)	9 (0–59)	0.94
FAC	0 (0–2)	0 (0–2.7)	0.78
MRC proximal upper limbs	2 (0–4)	2.5 (0–3)	0.99
MRC distal upper limbs	1 (0–4)	0.5 (0–3.5)	0.95
MRC proximal lower limbs	2 (1–4)	3 (2–4)	0.98
MRC distal lower limbs	1 (0–4)	1 (0–3)	0.34
MAS proximal upper limbs	0 (0–1)	0 (0–0)	0.3
MAS distal upper limbs	0 (0–0.5)	0.5 (0–3.5)	0.73
MAS proximal lower limbs	0 (0–1)	0 (0–1)	0.82
MAS distal lower limbs	0 (0–0.5)	0 (0–1)	0.36
**Angiogenin**			
Baseline levels (ng/mL)	502.7 ± 133.8	570.9 ± 145.8	0.09

*Variables are expressed as a percentage (number of cases), mean ± SD, or median (IQR). mRS, modified Rankin scale; BI, Granger modified Barthel Index; FMA, Fugl-Meyer Assessment; FAC, the Functional Ambulation Categories; CAHAI, Chedoke Arm and Hand Activity Inventory; MAS, Modified Ashworth Scale. Differences were assessed with a t-test, Mann-Whitney-U, chi-square test, or Fisher exact test*.

*Statistically significant differences are highlighted in bold*.

**Figure 2:**
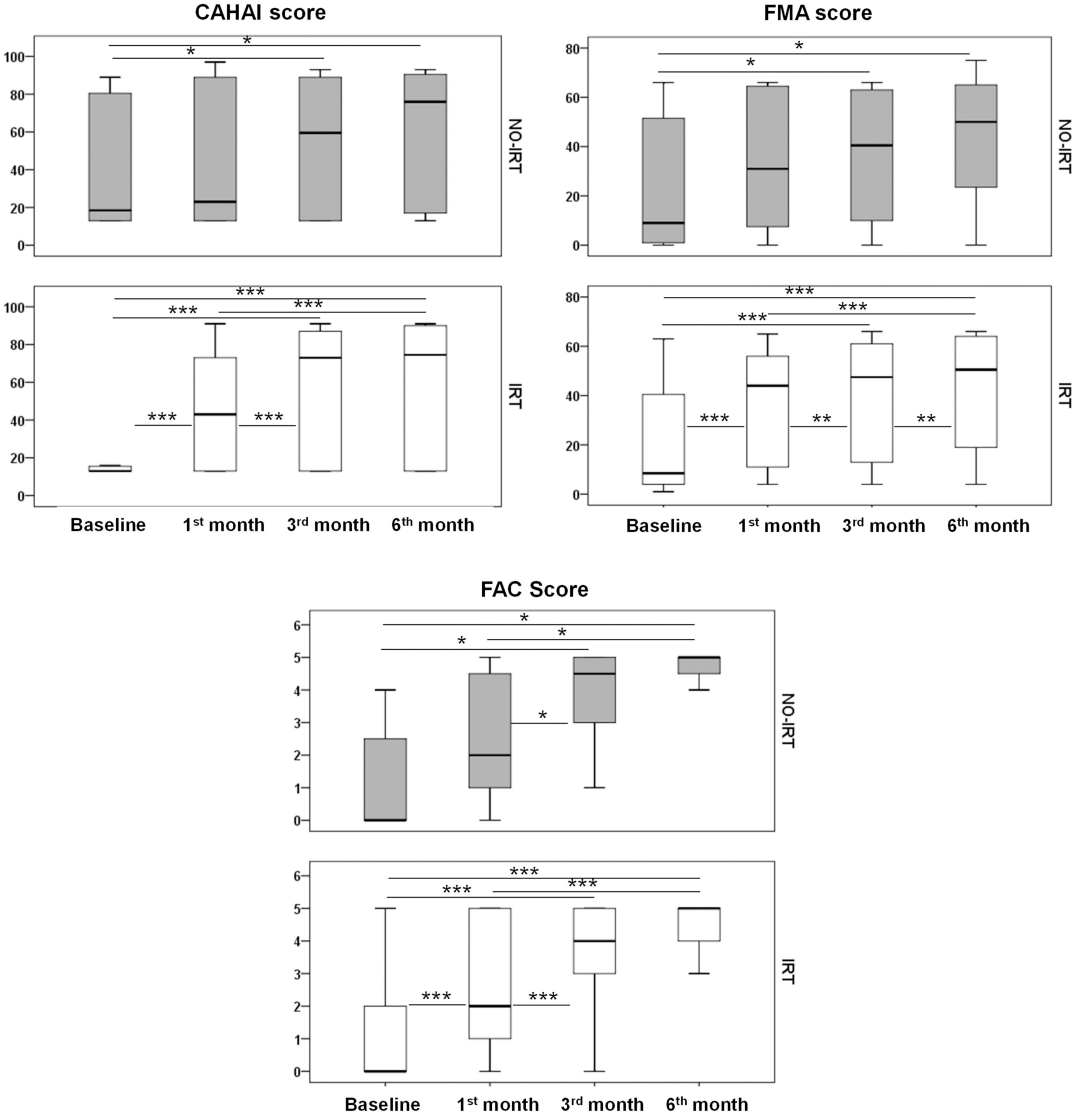
Functional outcome. Temporal profile of the tested scales in IRT and NO-IRT cohorts. Note that in the 1st month significant improvements were only achieved in the IRT group. Differences were assessed with the Wilcoxon tests and the Man-Withney U for the transversal analysis. ^*^p < 0.05; ^**^p < 0.01, and ^***^p < 0.001. Median and IQR are represented in box plots.

Both groups presented significant improvements over the 6 month follow-up period in functional and motor tests, but the CAHAI score and MAS scale only improved in the IRT group (Table 5). Regarding the impact of the rehabilitation intensity on motor and functional scores, Figure 2 shows improvements occurring earlier in the IRT group for the CAHAI, FMA, and FAC tests (*p* < 0.001 at 1 month), but not in the NO-IRT group, which achieved significance later at 3months (*p* < 0.05). Other tests presented similar profiles regardless of therapy intensity (Supplementary Figure 1). Importantly, at 3 months the IRT group achieved a full community ambulation level (0.89 m/s), but not the NO-IRT group (0.56 m/s) (Table 5). However we did not find differences overtime between rehabilitation groups in a mixed model: FAC (*p* = 0.86), Walking (*p* = 0.42), mRS (*p* = 0.82), BI (*p* = 0.41), FMA (*p* = 0.10), and CAHAI (*p* = 0.10). In this regard, the effect of time was independent of the type of therapy (*p* < 0.01) for all tests and did not change when adjusting for baseline mRS or Barthel.

**Table 5:** Measures of functional and motor outcome.

**IRT Group**	**Baseline**	**1st month**	**3rd month**	**6th month**
BI (0–100)^**^	35 (20–68)	77 (49–94)	93 (84–100)	100 (93–100)
mRS^**^	4 (3–5)	3 (2–4)	3 (1–3)	2 (1–2)
FMA (0–66)^*^	8.5 (4–40.7)	44 (9–56.5)	47.5 (12.5–61.3)	50.5 (17.8–64.5)
FAC (0–5)^**^	0 (0–2)	2 (1–5)	4 (3–5)	5 (4–5)
CAHAI (13–91)^**^	13 (13–15.7)	43 (13–76)	73 (13–87)	74 (13–90)
10-m walk test (m/s)^**^	NA	0.50 (0–1)	0.89 (0.25–1.22)	0.90 (0.54–1.30)
MRC scale superior-proximal (0–5)^**^	2 (0–1)	4 (2–4)	4 (2.5–5)	4 (2–5)
MRC scale superior-distal (0–5)^**^	1 (0–4)	2.5 (0–4)	4 (0.5–5)	4 (1–5)
MRC scale inferior-proximal (0–5)^**^	1 (0–4)	5 (4–5)	5 (4–5)	5 (4–5)
MRC scale inferior-distal (0–5)^**^	1 (0–4)	4 (1–5)	4 (2–5)	4 (2–5)
MAS scale superior-proximal (0–4)^**^	0 (0–1)	1 (0–1)	1 (0–2)	1 (1–2)
MAS scale superior-distal (0–4)^**^	0 (0–0.5)	1 (0–1.5)	1 (0–2)	1 (1–2)
MAS scale inferior-proximal (0–4)^**^	0 (0–1)	0 (0–1)	0 (0–1)	0 (0–1)
MAS scale inferior-distal (0–4)^**^	0 (0–0.5)	0 (0–1)	1 (0–2)	1 (0–2.25)
**NO-IRT Group**				
BI (0−100)^*^	23 (20–36.2)	49.5 (40–93)	88.5 (59–99)	90 (75–100)
mRS^**^	5 (3.2–5)	4 (1.2–4)	2.5 (1–3)	1 (1–3)
FMA (0–66)^*^	9 (0.5–55.2)	31 (7.2–64.7)	40.5 (9–63.5)	50 (18–66)
FAC (0–5)^*^	0 (0–2.7)	2 (1–4.75)	4.5 (3–5)	5 (4–5)
CAHAI (13–91)	18.5 (13–82.2)	23 (13–90)	59.5 (13–90)	76 (13–91)
10-m walk test (m/s)^**^	NA	0 (0–0.70)	0.56 (0.28–0.99)	0.95 (0.62–1.10)
MRC scale superior-proximal (0–5)^*^	2.5 (0–3)	3 (0–4)	3 (1.5–5)	4 (2–4)
MRC scale superior-distal (0–5)^*^	0.5 (0–3)	2.5 (0–4)	3.5 (1.5–4)	3.5 (1.5–5)
MRC scale inferior-proximal (0–5)^*^	1 (0–3)	4 (3–4)	4 (3.5–5)	4 (3.5–5)
MRC scale inferior-distal (0–5)^*^	1 (0–3)	2.5 (0–4)	4 (1–5)	4 (2–5)
MAS scale superior-proximal (0–4)	0 (0–0)	0.5 (0–1)	0 (0–1)	0 (0–1)
MAS scale superior-distal (0–4)	0 (0–0)	0.5 (0–1)	0.5 (0–1.75)	0 (0–2)
MAS scale inferior-proximal (0–4)	0 (0–0.75)	0 (0–1)	0.5 (0–1)	0 (0–1)
MAS scale inferior-distal (0–4)	0 (0–1)	0.5 (0–1)	1 (0–1.75)	0 (0–2)

*Data are shown as median (IQR). mRS, modified Rankin scale; BI, Granger modified Barthel Index; FMA, Fugl-Meyer Assessment; FAC, the Functional Ambulation Categories; CAHAI, Chedoke Arm and Hand Activity Inventory; MAS, Modified Ashworth Scale; NA, not applicable. Every scale shows the Median and IRQ Differences were assessed with a Friedman test:*

* *p < 0.05*,

** *p < 0.01*.

*Statistically significant differences are highlighted in bold*.

### Angiogenin Temporal Profile Changes With Therapy Intensity

**Figure 3:**
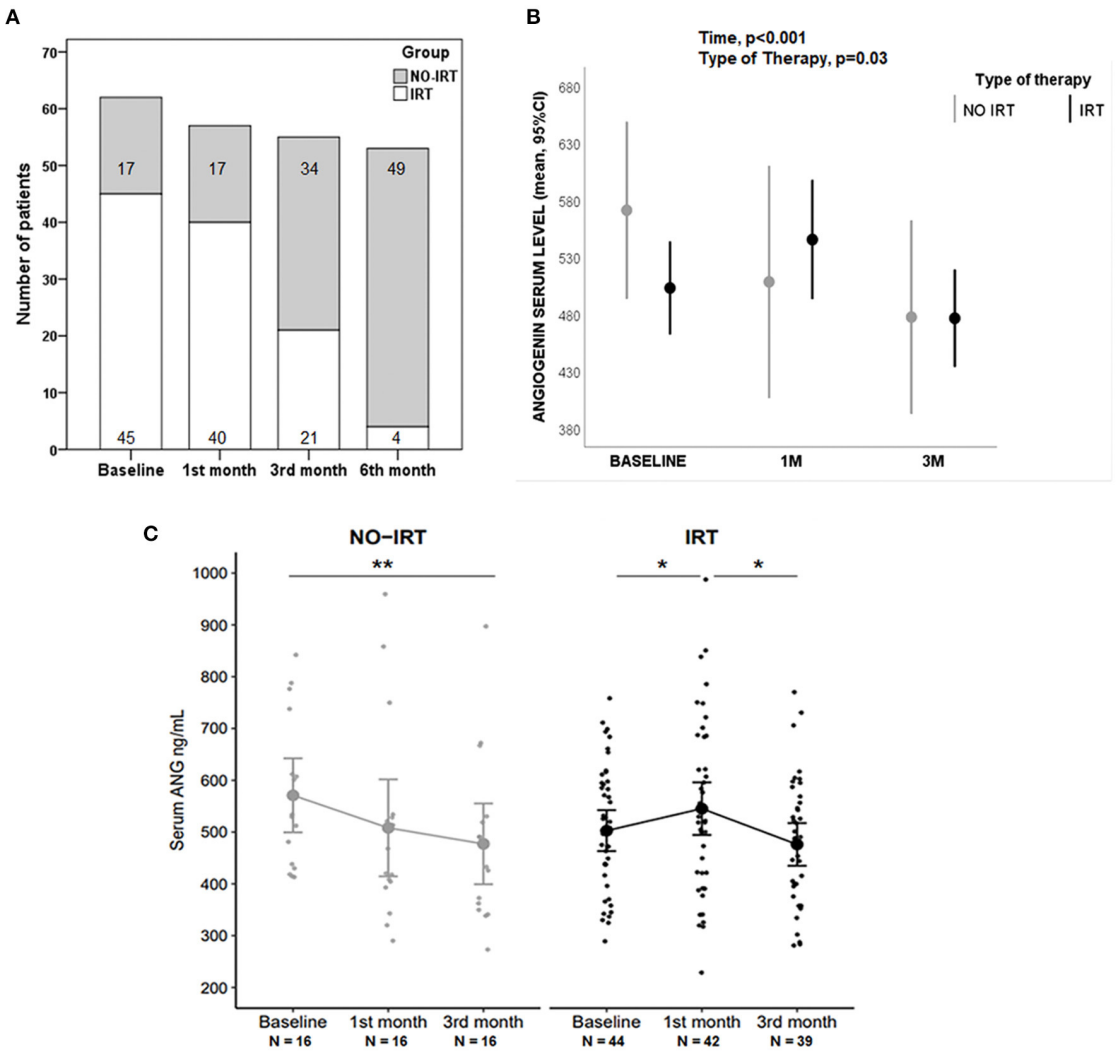
Angiogenin (ANG) blood levels during rehabilitation. **(A)** Bar graph showing the number of patients under IRT and NO-IRT over time, changing according to early improvements in the IRT group. **(B)** Graph comparing how time and type of therapy influenced the serum levels of ANG, assessed with ANOVA for repeated measures. **(C)** Jitter plots showing the temporal profile of ANG levels in NO-IRT and IRT groups; Differences were assessed with Wilcoxon tests, ^*^p < 0.05, ^**^p < 0.01. Mean with 95% CI are represented in graphs.

Baseline ANG was significantly higher in strokes than in controls (520 ± 139 vs. 432 ± 155 ng/mL; *p* < 0.01, see Table 1), with no baseline differences observed between the rehabilitation groups (see Table 4). No correlation was observed between baseline ANG and time-to-start RHB or baseline sampling. Notably, the number of rehabilitation hours were reduced according to the individual patient's achievements as represented in Figure 3A, with a substantial switch from IRT to NO-IRT over time. For this reason, the ANG level was analyzed only up to the 3rd month. In all strokes the ANG temporal profile showed significant differences (*p* < 0.001, see Supplementary Figure 2), being elevated at 1 month vs. controls (*p* = 0.036). Regarding the type of therapy, we found a significant interaction with ANG levels over time (*p* = 0.030, see Figure 3B). The ANG temporal profile in the NO-IRT and IRT groups showed differences (*p* < 0.01, see Figure 3C) in opposite directions since ANG increased after 1 month of IRT (*p* < 0.05 vs. baseline) but it decreased in the NO-IRT group (*p* < 0.01 vs. 3rd month).

Finally, we examined the relationship between outcome scores improvements from admission to 6 months follow–up with ANG, but we could not confirm a predictive value of ANG at any of the tested time points (data not shown).

## Discussion

The present study investigates the effects of rehabilitation therapy intensity on both functional outcome and blood levels of ANG, a potential biomarker of recovery. Our results suggest that stroke patients under more intense rehabilitation programs presented better outcomes earlier with a parallel increase of blood ANG. However, we could not confirm a predictive value of ANG.

Rehabilitation treatment is the gold-standard therapy for stroke survivors to recover functional status, and improve quality of life and independence (4) with programs personalized by multi-disciplinary teams, where the dose of the received therapy is a key factor in the recovery process (8, 13). In this study, we have followed two cohorts with similar clinical characteristics which primarily differed in the amount of scheduled therapy time at baseline. Our results suggest that patients under IRT receiving the highest therapy dose improved earlier at 1 month of therapy in the CAHAI and FMA scores, which according to the International Classification of Function (ICF-WHO) framework are designed to assess bilateral activity performance for daily life activities and brain structural recovery, respectively (29). Additionally, patients under IRT also achieved full community ambulation earlier during rehabilitation (30) and showed better BI scores earlier than NO-IRT patients, suggesting improved independence for the basic activities of daily living. In this regard and following previously reported dependence categories for the BI scale (31, 32), median BI values show that at 1 month IRT lead to a moderate dependence score whereas No-IRT maintained a severe dependence score and later at 3 months IRT lead to a slight independence score whereas No-IRT lead to a moderate dependence score.

Supporting these observations of larger functional gains in the IRT group, Wang and colleagues showed that the daily amount of physical, occupational, and speech and language therapy was also significantly associated with functional improvements (33). Others have previously reported a direct intensity-response between rehabilitation and functional recovery (8, 34). However, the patient's response to rehabilitation is heterogeneous and might depend on the type and amount of therapy received, or on individual endogenous neurorepair responses. In this regard, it is important to elucidate if IRT could further enhance recovery by increasing therapy intensity and/or duration.

A biomarker is an indicator that can be used to measure underlying molecular processes, identify a disease, predict recovery, or monitor treatment responses. In the present study, we show that only patients under IRT presented a significant increase in serum ANG after rehabilitation, which could be linked to the repair process underlying the prompt recovery since this angiogenic factor can trigger a wide range of biological processes (18). This improvement allowed the reduction of the amount of therapy time that paralleled a decrease of the serum ANG levels at the 3rd and 6th months of therapy. Whether extending the IRT therapy would maintain high ANG and result in larger functional/motor improvements remains to be elucidated in future interventional biomarker studies. Other studies have focused on identifying biomarkers for stroke disease (35, 36), but very few have focused on long-term outcomes or the influence of rehabilitation therapies. Others have explored the use of molecules related to oxidative stress (37) or changes in neurotransmitter levels during post-stroke rehabilitation, reporting a correlation with motor improvement (38). Serum ANG levels were first reported higher in patients with stroke within 48 h and on days 3 and 7, but decreasing at 14 days compared to control subjects (39), but the rehabilitation interventions were not described in this work. Our recent work also in serum ANG reported that blood ANG was increased in stroke patients after IRT (15), which is being confirmed in the present study with a larger cohort. Additionally, in this previous study, experiments in pre-clinical stroke models also described an ANG increase in the ischemic site when performing task-specific exercise, suggesting an association between rehabilitation and molecular changes in the brain. Moreover, another pre-clinical study from our group has recently described that physical exercise rapidly increased the amount of endogenous ANG in the ipsilateral neurogenic subventricular zone after cerebral ischemia (40). All this evidence points to potential connections between ANG, stroke disease, and rehabilitation, although confirmatory investigations are needed.

To our knowledge this is the first time a blood biomarker is described as differentially modulated by the amount of therapy time received during rehabilitation, suggesting the need to include rehabilitation treatments as a co-variable in stroke biomarker analyses, especially in multicenter studies.

As a limitation, our study presents a different number of patients in both rehabilitation cohorts due to different recruitment achieved by the participant centers, which could impact on group comparisons although baseline clinical and stroke characteristics are very similar. Also, in this observational study, the dose of therapy hours decreased over time in the IRT group based on clinical decisions and individual achievements, which could influence the levels of serum ANG between follow-up visits. For these reasons new interventional studies to determine the relationship between the dose of therapy and blood biomarkers are crucial to elucidate the true link with the clinical interventions.

In conclusion, our study shows that designing intensive rehabilitation programs results in earlier improvements, and the monitoring of specific blood biomarkers could be a useful part of the multidisciplinary recovery program.

## Data Availability Statement

Requests to access the anonymized datasets should be directed to the corresponding author, anna.rosell@vhir.org.

## Ethics Statement

The studies involving human participants were reviewed and approved by Research Ethics Committee sites [HUVH PR(IR)317/2013-PR(IR)346/2016, PI16/00981/CEI:PI-17-056, Comité de Ética de Investigación de A Coruña-Ferrol 2017-125, Corporació Sanitària Parc Taulí de Sabadell 2017521, Hospital Universitario Politécnico La Fe 2016/0727, Euskadi PI2016168]. The patients/participants provided their written informed consent to participate in this study.

## Author Contributions

NG-R, SR, XB, and AR participated in the design of the study. NG-R, SR, PT, ZM-A, XB, NR, MP-M, MB, JS, LP, MI, SO-V, RM-M, MM, MO-G, and AP participated in patients' recruitment and data collection. NG-R, XB, MQ, AP, and AR participated in data analysis. NG-R, SR, PT, ZM-A, NR, XB, MP-M, MB, JS, LP, MI, SO-V, RM-M, MM, MQ, MO-G, AP, and AR participated in the manuscript draft and/or review. All authors contributed to the article and approved the submitted version.

## Funding

NG-R holds a VHIR fellowship and MO-G a Joan Margarit VHIR fellowship. Research grants: from the Instituto de Salud Carlos III and European Regional Development Funds (PI16/00981, PI19/00186, RD16/0019/0021, and RD16/0019/0008), 2017-SGR-1427 program from the Generalitat de Catalunya-AGAUR, and Clinical Translational Program for Regenerative Medicine in Catalonia (P-CMR [C]).

## Conflict of Interest

LP and MI have received honoraria from Nestlé for activities unrelated to the present work. The remaining authors declare that the research was conducted in the absence of any commercial or financial relationships that could be construed as a potential conflict of interest.

## Publisher's Note

All claims expressed in this article are solely those of the authors and do not necessarily represent those of their affiliated organizations, or those of the publisher, the editors and the reviewers. Any product that may be evaluated in this article, or claim that may be made by its manufacturer, is not guaranteed or endorsed by the publisher.

## References

[1] Giroud M Jacquin A Béjot Y. The worldwide landscape of stroke in the 21st century. Lancet. (2014) 383:195–7. 10.1016/S0140-6736(13)62077-2 24449941

[2] Feigin VL Forouzanfar MH Krishnamurthi R Mensah GA Connor M Bennett DA. Global and regional burden of stroke during 1990–2010: findings from the Global Burden of Disease Study 2010. Lancet. (2014) 383:245–54. 10.1016/S0140-6736(13)61953-4 24449944 PMC4181600

[3] Licher S Darweesh SKL Wolters FJ Fani L Heshmatollah A Mutlu U. Lifetime risk of common neurological diseases in the elderly population. J Neurol Neurosurg Psychiatry. (2019) 90:148–56. 10.1136/jnnp-2018-318650 30279211

[4] Langhorne P Bernhardt J Kwakkel G. Stroke rehabilitation. Lancet. (2011) 377:1693–702. 10.1016/S0140-6736(11)60325-5 21571152

[5] AVERT Trial Collaboration Group. Efficacy and safety of very early mobilisation within 24 h of stroke onset (AVERT): a randomised controlled trial. Lancet. (2015) 386:46–55. 10.1016/S0140-6736(15)60690-0 25892679

[6] Bernhardt J Churilov L Ellery F Collier J Chamberlain J Langhorne P. Prespecified dose-response analysis for a very early rehabilitation trial (AVERT). Neurology. (2016) 86:2138–45. 10.1212/WNL.0000000000002459 26888985 PMC4898313

[7] Nakazora T Iwamoto K Kiyozuka T Arimoto H Shirotani T Domene K. Effectiveness of 7-day versus weekday-only rehabilitation for stroke patients in an acute-care hospital: a retrospective cohort study. Disabil Rehabil. (2018) 40:3050–3. 10.1080/09638288.2017.1367964 28826268

[8] Lang C Lohse K Birkenmeier R. Dose and timing in neurorehabilitation: prescribing motor therapy after stroke. Curr Opin Neurol. (2015) 28:549–55. 10.1097/WCO.0000000000000256 26402404 PMC4643742

[9] Imura T Nagasawa Y Fukuyama H Imada N Oki S Araki O. Effect of early and intensive rehabilitation in acute stroke patients: retrospective pre-/post-comparison in Japanese hospital. Disabil Rehabil. (2018) 40:1452–5. 10.1080/09638288.2017.1300337 28291953

[10] Lohse KR Lang CE Boyd LA. Is more better? Using metadata to explore dose-response relationships in stroke rehabilitation. Stroke. (2014) 45:2053–8. 10.1161/STROKEAHA.114.004695 24867924 PMC4071164

[11] Kamo T Momosaki R Suzuki K Asahi R Azami M Ogihara H. Effectiveness of intensive rehabilitation therapy on functional outcomes after stroke: a propensity score analysis based on japan rehabilitation database. J Stroke Cerebrovasc Dis. (2019) 28:2537–42. 10.1016/j.jstrokecerebrovasdis.2019.06.007 31235378

[12] Ward NS Brander F Kelly K. Intensive upper limb neurorehabilitation in chronic stroke: outcomes from the Queen Square programme. J Neurol Neurosurg Psychiatry. (2019) 90:498–506. 10.1136/jnnp-2018-319954 30770457

[13] Ballester BR Ward NS Brander F Maier M Kelly K Verschure PFMJ. Relationship between intensity and recovery in post-stroke rehabilitation: a retrospective analysis. J Neurol Neurosurg Psychiatry. (2021). 10.1136/jnnp-2021-326948. [Epub ahead of print]. 34168083 PMC8784991

[14] Krakauer JW Carmichael ST Corbett D Wittenberg GF. Getting neurorehabilitation right: what can be learned from animal models? Neurorehabil Neural Repair. (2012) 26:923–31. 10.1177/1545968312440745 22466792 PMC4554531

[15] Gabriel-Salazar M Morancho A Rodriguez S Buxó X García-Rodríguez N Colell G. Importance of angiogenin and endothelial progenitor cells after rehabilitation both in ischemic stroke patients and in a mouse model of cerebral ischemia. Front Neurol. (2018) 9:508. 10.3389/fneur.2018.00508 30008694 PMC6034071

[16] Korivi M Wu CY Lin KC. Potential predictive values of inflammatory biomarkers for stroke rehabilitation outcomes. J Formos Med Assoc. (2013) 112:735–7. 10.1016/j.jfma.2013.10.017 24326102

[17] Whiteley W Tian Y Jickling GC. Blood biomarkers in stroke: research and clinical practice. Int J Stroke. (2012) 7:435–9. 10.1111/j.1747-4949.2012.00784.x 22463131

[18] Goncalves KA Silberstein L Li S Severe N Hu MG Yang H. Angiogenin promotes hematopoietic regeneration by dichotomously regulating quiescence of stem and progenitor cells. Cell. (2016) 166:894–906. 10.1016/j.cell.2016.06.042 27518564 PMC4988404

[19] Sebastià J Kieran D Breen B King MA Netteland DF Joyce D. Angiogenin protects motoneurons against hypoxic injury. Cell Death Differ. (2009) 16:1238–47. 10.1038/cdd.2009.52 19444281

[20] Cuschieri S. The STROBE guidelines. Saudi J Anaesth. (2019) 13:S31–4. 10.4103/sja.SJA_543_18 30930717 PMC6398292

[21] Ictus: guia de pràctica clínica: actualització gener del 2007. 2a ed. Barcelona: Agència d'Avaluació de Tecnologia i Recerca Mèdiques (2007). Available online at: https://scientiasalut.gencat.cat/handle/11351/1853

[22] Mahoney FI Barthel DW. Functional evaluation. The Barthel Index. Maryland State Med J. (1965) 14:61–5. 10.1037/t02366-000 14258950

[23] Hernández ED Galeano CP Barbosa NE Forero SM Nordin Å Sunnerhagen KS. Intra- and inter-rater reliability of Fugl-Meyer Assessment of Upper Extremity in stroke. J Rehabil Med. (2019) 51:652–9. 10.2340/16501977-2590 31448807

[24] Barreca S Gowland CK Stratford P Huijbregts M Griffiths J Torresin W. Development of the Chedoke Arm and hand activity inventory: theoretical constructs, item generation, and selection. Top Stroke Rehabil. (2004) 11:31–42. 10.1310/JU8P-UVK6-68VW-CF3W 15592988

[25] Blackburn M van Vliet P Mockett SP. Reliability of measurements obtained with the modified Ashworth scale in the lower extremities of people with stroke. Phys Ther. (2002) 82:25–34. 10.1093/ptj/82.1.25 11784275

[26] Arya KN Verma R Garg RK. Estimating the Minimal clinically important difference of an upper extremity recovery measure in subacute stroke patients. Top Stroke Rehabil. (2011) 18:599–610. 10.1310/tsr18s01-599 22120029

[27] Barreca SR Stratford PW Lambert CL Masters LM Streiner DL. Test Retest reliability, validity, and sensitivity of the chedoke arm and hand activity inventory: a new measure of upper-limb function for survivors of stroke. Arch Phys Med Rehabil. (2005) 86:1616–22. 10.1016/j.apmr.2005.03.017 16084816

[28] Mehrholz J Wagner K Rutte K Meißner D Pohl M. Predictive validity and responsiveness of the functional ambulation category in hemiparetic patients after stroke. Arch Phys Med Rehabil. (2007) 88:1314–9. 10.1016/j.apmr.2007.06.764 17908575

[29] Yaruss JS Quesal RW. Stuttering and the international classification of functioning, disability, and health: an update. J Commun Disord. (2004) 37:35–52. 10.1016/S0021-9924(03)00052-2 15013378

[30] Perry J Garrett M Gronley JK Mulroy SJ. Classification of walking handicap in the stroke population. Stroke. (1995) 26:982–9. 10.1161/01.STR.26.6.982 7762050

[31] Ploughman M Beaulieu S Harris C Hogan S Manning OJ Alderdice PW. The Canadian survey of health, lifestyle and ageing with multiple sclerosis: methodology and initial results. BMJ Open. (2014) 4:e005718. 10.1136/bmjopen-2014-005718 25011993 PMC4120418

[32] Patricia P. Measures of adult general functional status: the Barthel index, Katz index of activities of daily living, health assessment questionnaire (HAQ), MACTAR patient preference disability questionnaire, and modified health assessment questionnaire (MHAQ). Arthritis Rheum. (2003) 49:S15–27. 10.1002/art.1141525855820

[33] Wang H Camicia M Terdiman J Mannava MK Sidney S Sandel M. Daily treatment time and functional gains of stroke patients during inpatient rehabilitation. PM R. (2013) 5:122–8. 10.1016/j.pmrj.2012.08.013 23122894

[34] Jette DU Warren RL Wirtalla C. The relation between therapy intensity and outcomes of rehabilitation in skilled nursing facilities. Arch Phys Med Rehabil. (2005) 86:373–9. 10.1016/j.apmr.2004.10.018 15759214

[35] Montaner J Ramiro L Simats A Tiedt S Makris K Jickling GC. Multilevel omics for the discovery of biomarkers and therapeutic targets for stroke. Nat Rev Neurol. (2020) 16:247–64. 10.1038/s41582-020-0350-6 32322099

[36] Ramiro L Simats A García-Berrocoso T Montaner J. Inflammatory molecules might become both biomarkers and therapeutic targets for stroke management. Ther Adv Neurol Disord. (2018) 11:1756286418789340. 10.1177/1756286418789340 30093920 PMC6080077

[37] Hsieh YW Lin KC Korivi M Lee TH Wu CY Wu KY. The reliability and predictive ability of a biomarker of oxidative DNA damage on functional outcomes after stroke rehabilitation. Int J Mol Sci. (2014) 15:6504–16. 10.3390/ijms15046504 24743892 PMC4013643

[38] Blicher JU Near J Næss-Schmidt E Stagg CJ Johansen-Berg H Nielsen JF. GABA levels are decreased after stroke and GABA changes during rehabilitation correlate with motor improvement. Neurorehabil Neural Repair. (2015) 29:278–86. 10.1177/1545968314543652 25055837 PMC5435106

[39] Huang L Guo H Cheng M Zhao Y Jin X. The kinetic change of the serum angiogenin level in patients with acute cerebral infarction. Eur Neurol. (2007) 58:224–7. 10.1159/000107944 17823536

[40] Gabriel-Salazar M Lei T Grayston A Costa C Medina-Gutiérrez E Comabella M. Angiogenin in the neurogenic subventricular zone after stroke. Front Neurol. (2021) 12:662235. 10.3389/fneur.2021.662235 34234733 PMC8256153

