# Supplementary material for: Functional Recovery and Serum Angiogenin Changes According to Intensity of Rehabilitation Therapy After Stroke
Source: Front Neurol. 2021 Nov 25;12:767484. doi: 10.3389/fneur.2021.767484 (PMC8655101; doi:10.3389/fneur.2021.767484)

SUPPLEMENTARY FIGURES

Functional recovery and serum angiogenin changes according to intensity of rehabilitation therapy after stroke.

Nicolás Garcia-Rodriguez, MD[1,2], Susana Rodriguez, MD[2], Pedro Ignacio Tejada, MD[3], Zuberoa Maite Miranda-Artieda, MD[3], Natalia Ridao, MD[4], Xavi Buxó, MD[2], María Engracia Pérez-Mesquida, MD[2], Maria Rosario Beseler, MD[5], Juan B. Salom, MD[6,7], Laura M. Pérez, MD[8,9], Marco Inzitari, MD[8,10], Sergio Otero-Villaverde, MD[11], Rosa Martin-Mourelle, MD[11], Mercedes Molleda, MD[12], Manuel Quintana, MSc[13], Marta Olivé-Gadea, MD[14], Anna Penalba[1], Anna Rosell, PhD[1]

[1]Neurovascular Research laboratory, Vall d'Hebron Research Institute, Universitat Autònoma de Barcelona. Spain.

[2]Unidad de Rehabilitación Neurológica y Daño Cerebral, Hospital Vall d'Hebron, Barcelona, Spain.

[3]Unidad de Daño Cerebral del Hospital de Górliz. Osakidetza. Spain.

[4]Servei de Medicina Física i Rehabilitació. Parc Taulí Hospital Universitari. Institut d'Investigació i Innovació Parc Taulí I3PT. Sabadell. Spain.

[5] Servicio de Medicina Física y Rehabilitación, Hospital Universitario y Politécnico La Fe, Valencia, Spain.

[6]Unidad Mixta de Investigación Cerebrovascular, Instituto de Investigación Sanitaria La Fe–UV, Valencia, Spain.

[7]Departamento de Fisiología, Universidad de Valencia, Valencia, Spain.

[8] RE-FiT Barcelona Research Group, Vall d'Hebron Institute of Research and Parc Sanitari Pere Virgili, Barcelona, Spain.

[9]Parc Sanitari Pere Virgili, Area of Intermediate Care, Barcelona, Spain.

[10]Universitat Oberta de Catalunya.

[11]Hospital Marítimo de Oza, A Coruña, Spain.

[12]Hospital Universitari Germans Tries i Pujol, Badalona, Spain.

[13]Epilepsy Unit and Research Laboratory Hospital Vall d'Hebron, Barcelona, Spain.

[14]Stroke Group. Vall d'Hebron Research Institute, Barcelona, Spain.

Corresponding author: Anna Rosell, PhD. Neurovascular Research Laboratory, Institut de Recerca Hospital Vall d'Hebron. Passeig Vall d'Hebron 119-129, Barcelona, Spain. +34 93489409; anna.rosell@vhir.org

Figure S1. Functional outcome. Box-plots representing the median and IQR temporal profile of the tested scales during follow-up in IRT and NO-IRT cohorts. Differences were assessed with the Wilcoxon test, *p<0.05; **p<0.01, and ***p<0.001.

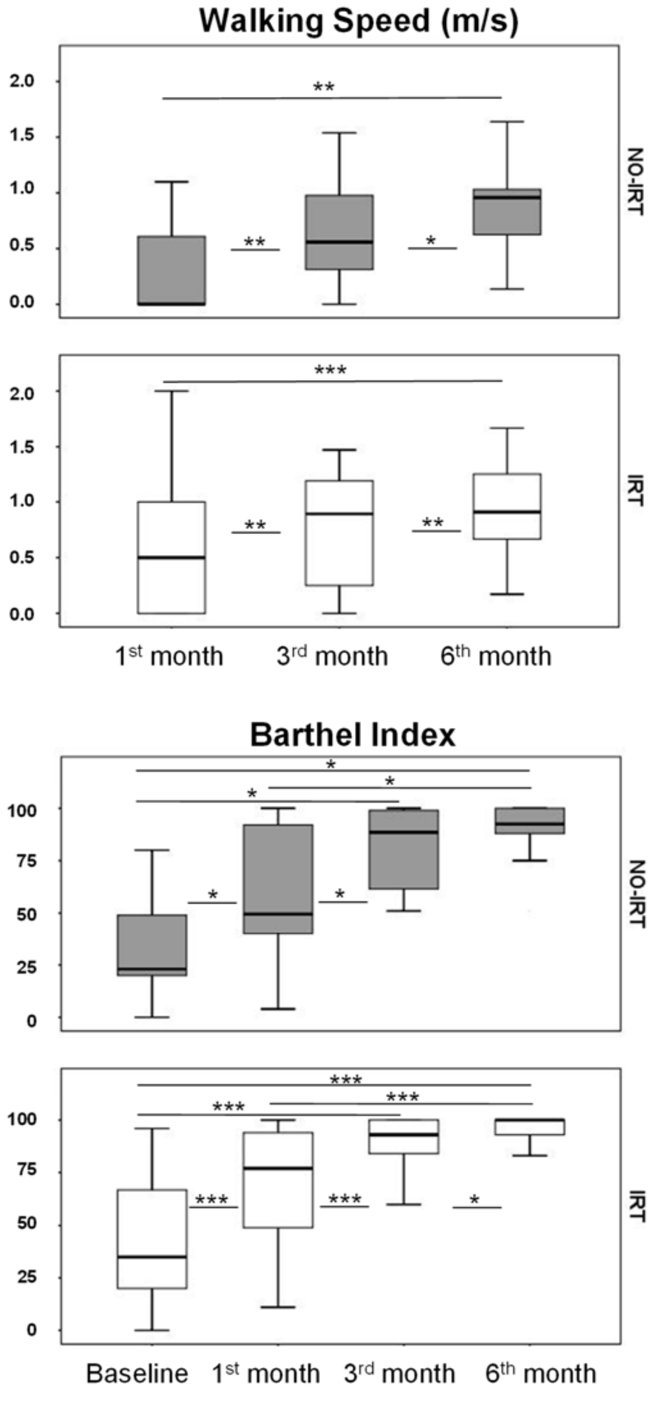

Figure S2. Serum Angiogenin. Jittler plots showing the temporal profile of serum angiogenin levels in all strokes regardless the type of rehabilitation therapy. Bars represent mean and 95%CI, and the dashed line shows mean Control levels. Differences were assessed with the Wilcoxon test, *p<0.05.

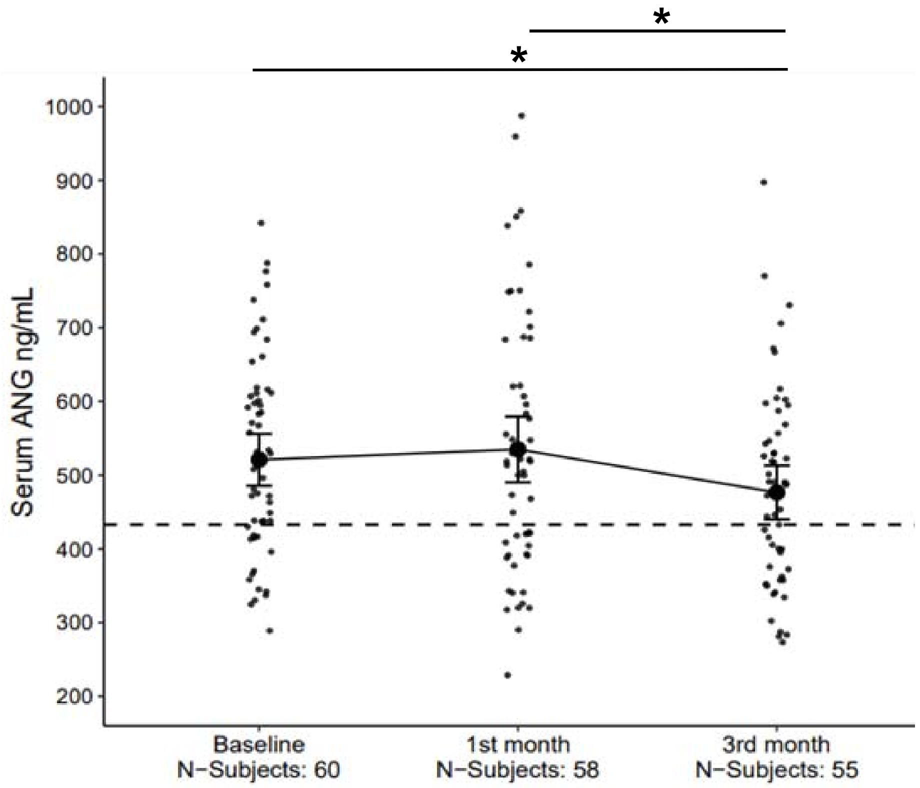

Supplement: Supplementary file 1 [file Data_Sheet_1.pdf]
